# Role of Milk-Derived Opioid Peptides and Proline Dipeptidyl Peptidase-4 in Autism Spectrum Disorders

**DOI:** 10.3390/nu11010087

**Published:** 2019-01-04

**Authors:** Beata Jarmołowska, Marta Bukało, Ewa Fiedorowicz, Anna Cieślińska, Natalia Karolina Kordulewska, Małgorzata Moszyńska, Aleksander Świątecki, Elżbieta Kostyra

**Affiliations:** 1 Department of Biochemistry, Faculty of Biology and Biotechnology, University of Warmia and Mazury, Oczapowskiego 1A Street, 10-19 Olsztyn, Poland; bj58@wp.pl (B.J.); marta.bukalo@gmail.com (M.B.); ewa.kuzbida@uwm.edu.pl (E.F.); natalia.smulska@uwm.edu.pl (N.K.K.); elzbieta.kostyra@uwm.edu.pl (E.K.); 2 Center for Diagnosis, Treatment and Therapy of Autism at the Regional Children’s Hospital in Olsztyn, Zolnierska 18 A Street, 10-561 Olsztyn, Poland; magmoszyn@gmail.com; 3 Faculty of Biology and Biotechnology, Department of Microbiology and Mycology, University of Warmia and Mazury, Oczapowskiego 1A Street, 10-19 Olsztyn, Poland; aswiat@uwm.edu.pl

**Keywords:** autism spectrum disorder, β-casomorphin-7, bovine milk, food allergy, proline dipeptidyl peptidase-4

## Abstract

Opioid peptides released during digestion of dietary proteins such as casein, were suggested to contribute to autism development, leading to the announcement of opioid excess hypothesis of autism. This paper examines role of enzyme proline dipeptidyl peptidase-4 (DPPIV; EC 3.4.14.5) and it is exogenous substrate, β-casomorphin-7 (BCM7) in autism etiology. Our study included measurements of DPPIV and BCM7 concentrations in serum and urine, which were analyzed with ELISA assays and activity of DPPIV was measured by colorimetric test. The effect of opioid peptides from hydrolysed bovine milk on DPPIV gene expression in peripheral blood mononuclear cells (PBMC) in autistic and healthy children was determined using the Real-Time PCR (Polymerase Chain Reaction) method. Our research included 51 healthy children and 86 children diagnosed with autism spectrum disorder (ASD, ICDF84). We determined that the concentration of BCM7 in serum was significantly, 1.6-fold, higher in the ASD group than in controls (*p* < 0.0001). Concentration of DPPIV was found to also be significantly higher in serum from ASD children compared to the control group (*p* < 0.01), while we did not notice significant difference in enzymatic activity of serum DPPIV between the two study groups. We confirmed correlation according to the gender between analyzed parameters. The inspiration for this study emanated from clinical experience of the daily diet role in relieving the symptoms of autism. Despite this, we have concluded that milk-derived opioid peptides and DPPIV are potentially factors in determining the pathogenesis of autism; conducted studies are still limited and require further research.

## 1. Introduction

Autism Spectrum Disorders (ASDs) are the pervasive developmental disorders characterized by repetitive, stereotyped patterns of behavior, impaired social interaction, and communication. ASDs are a combination of complex neurological developmental disorders, which include autistic disorder, Asperger’s syndrome, and pervasive developmental disorders (PDDs), are not otherwise specified [1]. During the last 40 years, there has globally been 20–30-fold increases in the prevalence of autism. The prevalence of autism spectrum disorders varies worldwide from 1.4/10,000 children in the Arabian Peninsula to 185/10,000 children of Asian population. In Europe, the highest prevalence has been observed in Sweden (115/10,000), while the lowest has been in Croatia (2–3/10,000, respectively) [2]. The occurrence of autism is ranging from three-to-five times higher in males than females [3]. While this asymmetry in the male-female ratio has been known for many years, the underlying factors remain largely unknown and this proportion is actually broadly revised [4]. Nowadays, ASD is considered as a multifactorial disease, promoted by a variety of genetic and environmental factors [5,6]. 

It has been widely discussed that individuals with ASD may have undiagnosed gastrointestinal conditions and hypersensitivities, which are the effects of the ingestion of casein and gluten. This disorders may result in an exacerbation of ASD behaviors (e.g., tantrums, screaming, and aggression) and inattention to tasks due to the distraction, and because of the pain [7]. According to the previous hypothesis postulated by Panksepp, disruptions in opioid system are possible because of the so-called autistic behavior [8] and this opioid theory of autism was also proved by pharmacological studies [9]. Exogenous opioid peptides released during the digestion of dietary proteins, such as casein and gluten were suggested to contribute to autism development, leading to the announcement of the opioid excess hypothesis of autism [10]. 

One of the most biologically active milk-derived peptides are β-casomorphins (BCMs), in particular, β-casomorphin-7 (BCM7; YPFPGPI) released from bovine β-casein sequence [11]. BCM7 has the ability to permeate the intestinal barrier and may induce biological effects through the μ-opioid receptors (MORs) in the immune and nervous systems [12,13]. Moreover, BCMs were found to have an inflammatory effect on the gastrointestinal system and may contribute to etiology of food allergy [12,14]. In vitro studies have shown that BCM7 may alter lymphocyte proliferation and inflammatory markers release [14,15]. Elevated levels of BCM7 have been observed in serum and urine of ASDs patients in several independent studies [16,17]. The presence of milk-derived opioid peptides is confirmed in bovine milk [11], breast milk and infant formula [18,19,20], dairy products including cheese or yoghurt [13,18,21], and high-protein supplements for sportsmen [22]. 

The concentration of milk-derived opioid peptides like casomorphins in the human body depends on several factors: The content in the food, the release from the protein precursors during food digestion, and proline dipeptidyl peptidase-4 activity (DPPIV, CD26; EC 3.4.14.5). BCMs are identified as exogenous substrates for DPPIV and inactivated under the influence of the enzyme. Deficiency of DPPIV and/or its lower enzymatic activity were suggested as possible causes for the increased level of opioids in patients with autism [23,24]. Moreover, DPPIV is involved in immune response and nonspecific inflammatory processes and its decreased activity is generally associated with impaired immune status [25]. 

Milk-derived opioid peptides should be considered as environmental stressors, which can lead to disorders in the gut, reduced proteolytic activity, and increased permeability of the intestinal barrier. These aspects, in correlation with low levels of circulating peptidases and increased blood–brain barrier permeability, may cause the accumulation of opioid peptides in the blood and the brain [17]. The consequence is opioid-dependent modulation of neurotransmitter system and central nervous system functioning, which may lead to the development of ASD [26]. Therefore, a currently proposed treatment is the gluten-free, casein-free (GFCF) diet, supported by numerous scientific reports [27,28,29], and information obtained from the parents about a significant improvement in health and even recovery of children with symptoms of ASD [30]. 

The aim of this study was determination of BCM7 influence on DPPIV functioning in children with ASD in comparison to healthy children, which is also according to gender. For this purpose, we examined content and activity of serum DPPIV, content of BCM7 in serum and urine, and studied the effect of hydrolysed bovine milk, as a source of opioid peptides, on DPPIV gene expression in peripheral blood mononuclear cells (PBMC) in both groups.

## 2. Materials and Method

### 2.1. Patients and Control Group Characteristic

Our research included 86 children diagnosed with autism spectrum disorder (ASD, ICDF84) (71 male and 15 female; age range 3–10 years; and age mean 5.4 years), considered in this paper as research group. The control group consisted of 51 healthy children with no history of behavioral disorders (32 male and 19 female; age range 3–9 years; and age mean 5.2 years). The patients were recruited by specialists in the Center for Diagnosis, Treatment and Therapy of Autism at the Regional Children’s Hospital in Olsztyn, Poland. Diagnosis was based on the International Classification of Mental and Behavioural Disorders—ICD-10. F84 disease in children was identified on the basis of interdisciplinary differential diagnosis: Psychiatric examination excluding mental illness; studies evaluating cognitive parameters in the respondents (Leiter scale—standard IQ from 70 to 107; Wechsler—standard IQ from 90 to 104); neurological examination—EEG, evaluation of reflexes; speech therapy—evaluation study of the development of speech; passive and participatory observation lasting from 6 to 12 months; and analysis of the documentation: Names of parents, the opinions of educational institutions, and video. Subjects with fever, infections, or skin problems and those taking steroids or antibiotics were excluded from the study or control group. All subjects gave their written informed consent for inclusion before they participated in the study. The study was approved by the Local Bioethics Committee (number 19/2016 from the date of 18 May 2016).

### 2.2. Examined Substances

The study used pasteurized bovine milk which was commercially available. Prior to the experiments, milk was hydrolyzed enzymatically to reflect human food digestion. Enzymatic hydrolysis and sample preparation were previously described and published by Fiedorowicz et al. [12]. In brief, 20 mL of hydrolysed milk was acidified to pH 4.2, centrifuged at 10,000× *g*, 25 min, and 4 °C. The middle layer was collected and ultrafiltrated using 30 kDa and 3 kDa cut-off points membrane tubes (AmiconUltra 30 and 3 kDa, Millipore, Burlington, MA, USA) in the following conditions: 5000× *g*, 30 min, 4 °C. The peptide extract obtained from hydrolyzed bovine milk was immediately used for analyses or stored at −80 °C. Before the in vitro analysis sample was diluted 2.5 times. Examined substances were sterilized through a 0.22 μm filter (Becon Dickinson, Franklin Lakes, NJ, USA). 

### 2.3. Biological Material

One of the 5–10 mL peripheral blood samples, 2 mL of serum, and 15 mL of urine were collected from each patient. Samples were obtained by medical staff at the Regional Children’s Hospital in Olsztyn. Biological material was immediately transported to the laboratory and was used for analysis or stored at −80 °C.

### 2.4. Measurement of BCM7 Concentration

ELISA test enabled identification of BCM7 contents in the serum and urine from patients, as well as in tested peptide extract obtained from hydrolyzed bovine milk. The analysis was performed in triplicate by means of the method previously described [13]. The results were analyzed in the GraphPad PRISM version 6.0 (GraphPad Software, Inc., La Jolla, CA, USA) and the BCM7 concentrations were determined, based on the standard curve for the concentrations 0.001–100 μg/mL.

### 2.5. Measurement of DPPIV Activity and Concentration in Serum

DPPIV activity was determined with the “direct photometric method”, adapted to 96-well plates, as was described by Jarmołowska et al. [20]. Briefly, performance of analysis was as follows: 10 μL of test serum, water (blank) or standard (3 mM p-nitroanilide) was added to the reaction mixture contained 50 μL 0.3 M Gly/NaOH buffer (pH 8.7), 100 μL 3 mM Gly-Pro-p-nitroanilide p-toluenosulfonate and 50 μL water. Control wells had no serum. After 30 min of incubation at 37 °C, the reaction was stopped by adding 50 μL ice-cold (4 °C) 1 M acetate buffer (pH 4.2) and 10 μL of test serum was added to the control wells. The absorbance was measured at 405 nm with a microplate reader (Asys UVM 340, Biochrom, Holliston, MA, USA). The calculations were made after adjusting the measurements with the blank. The enzyme activity was calculated as: 100 × (E − C)/S (where E, C, and S stand for the absorbance of the test, control and standard samples, respectively). One unit of the enzyme activity was defined as the amount of the enzyme liberating 1 μmol p-nitroanilide/min/L test serum at 37 °C.

Concentration of DPPIV in serum were evaluated using commercial ELISA kit (BioVendor, Brno, Czech Republic) according to the manufacturer instructions.

### 2.6. Analysis of the DPPIV Gene Expression under the Influence of Peptide Extract Obtained from Hydrolysed Bovine Milk

The blood from the patients was obtained directly into tubes containing K_3_ETDA (BD, Biosciences) and the peripheral blood mononuclear cells (PBMCs) isolation was immediately started. Fresh PBMCs were then prepared, as previously described by Fiedorowicz et al. [15]. PBMCs were counted by automatic cell counter—Scepter (MerckMillipore). The cells were seeded into 24-well plates at 0.5 × 10^6^ per well with RPMI-1640 (Sigma, St. Louis, MO, USA) and supplemented with 1% heat inactivated human AB serum (Sigma), 1% gentamicin (Sigma) and 0.25% phytohaemagglutinin (PHA, Roche, Basel, Switzerland). After 48 h incubation, 300 μL peptide extracts obtained from hydrolyzed bovine milk was added to each well, giving a final 5-fold dilution with the culture medium. The incubation with the examined substance was conducted for five days. PBMCs suspension was centrifuged (800× *g*, 20 °C, 5 min), cell residue was rinsed twice with Dulbecco’s Phosphate-Buffered Saline (DPBS, Invitrogen, Carlsbad, CA, USA) and used for RNA isolation.

RNA was isolated from the PBMCs using TRIzol Reagent (Sigma, St. Louis, USA), as previously described by Kordulewska et al. [31]. RNA purity was estimated by calculating the ratio between absorbance at 260 and 280 nm (A260/A280), with 1.8–2.0 results, and stored at −80 °C for further analysis. The reaction of reverse transcription was carried out using the QuantiTect Reverse Transcription set (Qiagen, Hilden, Germany) according to the producer instructions. The obtained cDNA was used as a matrix for the qualitative identification of gene amplification in Real Time PCR. The 7500 FAST Sequence Detection System equipment and Fast SYBR Green Master Mix reader by Applied Biosystems (Foster City, CA, USA) were used for the analysis. DPPIV gene and the housekeeping human β-actin gene (ACTB) were examined, with ACTB used as a reference gene to normalize differences in total RNA amounts in each sample. Oligonucleotide primers specific to each gene were designed with Primer-BLAST, and the PCR primers are listed in Table 1.

Reaction was carried out in the following conditions: preliminary polymerase activation 95 °C, 20 s; 40 cycles: 3 s, 95 °C; 60 °C/ACTB/DPPIV, 30 s. The control of the product purity was performed with the use of electrophoresis in 1.5% agarose gel (Sigma). The results were presented in relative units in relation to the level of gene expression in native cells, referred to as 1 using the method described by Pfaffl [32].

### 2.7. Statistical Data

All statistical analyses were performer using GraphPad Prism 6.0 software (GraphPad Software Inc., San Diego, CA, USA). Results have been presented as a mean ± SEM. Student’s *t*-test was used for comparison of parametric data—DPPIV activity in serum, while Mann-Whitney U-test for content DPPIV in serum. Kruskal-Wallis test was used for comparison of non-parametric data. Gene expression gender differences were analyzed using a two-way Anova test. Correlations were tested using the Spearman test. 

## 3. Results

### 3.1. Measurement of BCM7 Concentration in Hydrolysed Bovine Milk and Patients’ Serum and Urine

Application of the ELISA test confirmed the presence of BCM7 in peptide extract obtained from hydrolyzed bovine milk. The mean concentration of BCM7 in the sample was 60 ng/mL (data not shown).

**Figure 1:**
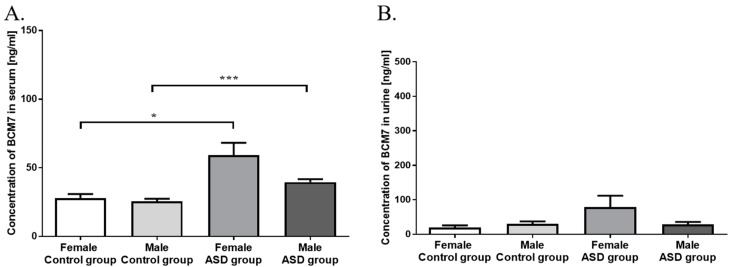
Comparison of serum (**A**) and urine (**B**) BCM7 concentration in control and autistic group (ASD) (ng/mL ± SEM). Significant differences between groups, divided by gender: * *p* < 0.05; *** *p* < 0.001.

We have analyzed a concentration of BCM7 in serum, which was more significantly different (*p* < 0.0001) in the ASD group (42.96 ± 2.52 ng/mL) than in controls (26.42 ± 1.63 ng/mL) (Figure 1A). We showed that the higher concentrations of serum BCM7 (*p* < 0.001) occurred among ASD boys (39.52 ± 2.26 ng/mL) compared to control boys (25.5 ± 1.9 ng/mL). We also noted significant difference (*p* < 0.05) occurred among ASD girls (59.2 ± 9.04 ng/mL) than in control girls (27.9 ± 2.97) (Figure 1A).

Urine BCM7 concentration is presented in Figure 1B. There were no significant difference detected in urine levels of BCM7 between two study groups, and also between gender (Figure 1B).

### 3.2. Measurement of DPPIV Activity and Concentration in Serum

**Figure 2:**
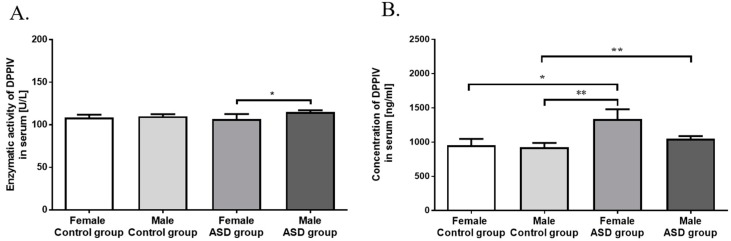
Comparison of DPPIV enzymatic activity (**A**) and concentration (**B**) in serum of control and autistic group (ASD) (Figure 2A: U/L ± SEM; Figure 2B: ng/mL ± SEM). Significant differences between groups, divided by gender: * *p* < 0.05, ** *p* < 0.01.

Figure 2A,B show serum DPPIV activity in control and ASD groups. We did not notice significant difference in enzymatic activity of serum DPPIV between two study groups (*p* = 0.262; Figure 2A). The significantly lower DPPIV activity (*p* < 0.05) was observed in girls with autism, compared to ASD boys (Figure 2A).

DPPIV concentration was found significantly higher (*p* < 0.01) in serum from ASD children (1089 ± 44.3 ng/mL) compared to the control group (934.0 ± 52.7 ng/mL) (Figure 2B). The concentration of serum DPPIV was significantly higher (*p* < 0.01) in autistic (1050 ± 36.86 ng/mL) than in healthy boys (922.2 ± 65.04 ng/mL). There was also statistical difference between the concentration of serum DPPIV in ASD girls (1336 ± 144.0 ng/mL) compared to both control girls (953.4 ± 92.14 ng/mL, *p* < 0.05) and control boys (*p* < 0.01) (Figure 2B).

### 3.3. DPPIV Gene Expression in PBMCs

**Figure 3:**
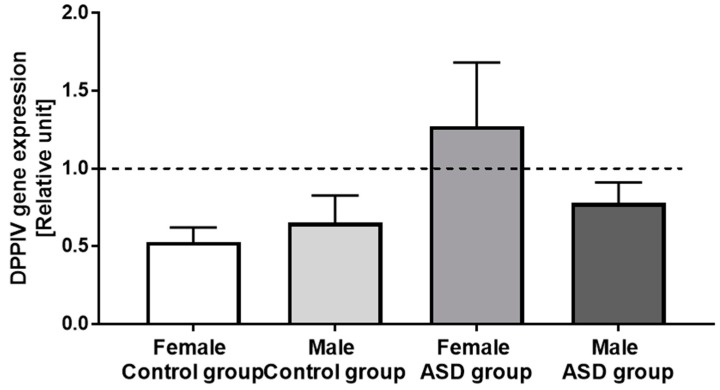
Influence of milk-derived opioid peptides on DPPIV gene expression in control and autistic group (ASD), and divided by gender (relative unit ± SEM).

There were no significant differences in DPPIV gene expression under the influence of extract obtained from hydrolyzed bovine milk. There were also no differences according to gender. Nevertheless, we noticed a tendency to increase the DPPIV gene expression among girls with autism (Figure 3).

### 3.4. Correlation of Examined Parameters between ASD and Control Groups, and with Gender

**Figure 4:**
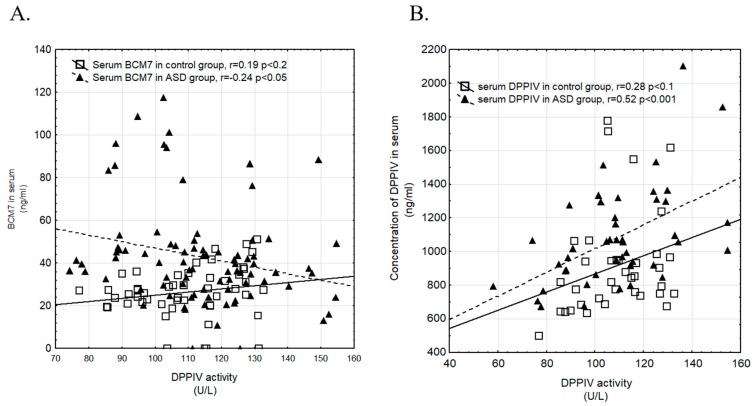
Correlation between DPPIV activity and BCM7 concentration in serum (**A**) and between DPPIV activity and concentration in serum (**B**) in control and ASD group (▲—ASD group, □—control group).

Figure 4A,B show correlation between DPPIV activity and BCM7 concentration (Figure 4A), and DPPIV concentration and activity in ASD and control groups (Figure 4B). We observed a negative correlation between concentration of BCM7 and DPPIV activity in serum in the ASD group (*p* < 0.05). Positive correlation between concentration and activity DPPIV in serum was observed in both, control (*p* < 0.01) and ASD group (*p* < 0.001). 

**Figure 5:**
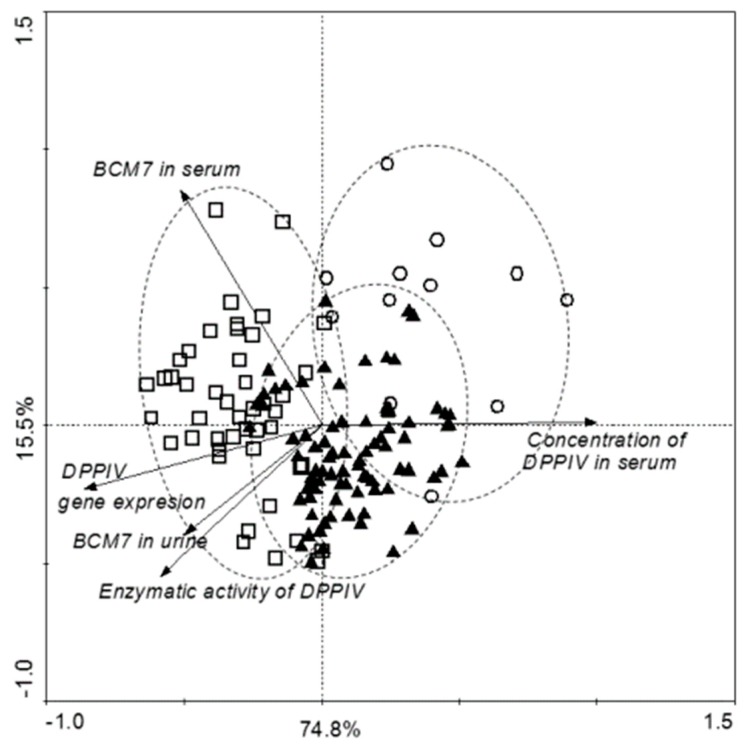
Correlations between all tested parameters in control and autistic group (ASD), and divided by gender (▲—boys with ASD, ○—girls with ASD, □—healthy children).

Figure 5 summarizes all obtained results. Principal component *analysis* (PCA) showed significant differences within the tested samples. Concentration of DPPIV and BCM7 in serum was statistically significantly higher in autistic children than in the control group. According to the gender correlation found between DPPIV activity and BCM7 concentration in serum (R = 0.418, *p* = 0.075), and DPPIV activity and concentration in serum (R = 0.503, *p* = 0.067) in healthy girls, and between DPPIV activity and DPPIV concentration in serum in ASD boys (R = 0.446, *p* = 0.005). A particularly interesting are ASD girls, characterized by the correlation between the concentration of BCM7, DPPIV content, and DPPIV expression.

## 4. Discussion

Autism is a developmental disorder, which usually manifests itself in the first years of life. Children with autism exhibit abnormal behavior in social interaction and communication problems. As indicated by the global statistics from year-to-year, the incidence of autism increases and is currently epidemic of this disease should be considered [1,33]. ASD prevalence is strongly associated with the male gender [3], which is also represented by our sex distribution in the ASD group. The causes of autism are not fully explained, but it is clear that the development of the disease is affected by genetic and autoimmune factors, metabolic disorders, and epigenetic changes depending on the environmental and nutritional factors. In recent years, researchers have focused on the role of the opioid system in various pathological processes [33].

Numerous studies confirmed, that milk-derived opioid peptides may penetrate the intestinal barrier and induce biological effects through the opioid receptors of the immune and nervous system [12,34,35]. We found that the content of BCM7 in serum was significantly higher (*p* < 0.0001) in ASD than in the control group (Figure 1A). Elevated level of BCM7 in serum of autistic children was reported by several authors [9,17], which is consistent with our results. We also noticed a difference between gender—in ASD girls there was the highest level of BCM7 in serum (*p* < 0.05) among all patients (Figure 1A). Statistical difference between ASD girls and boys can be explained as small number of girls. However, obtaining a similar number of girls and boys in ASD group was difficult because it is associated with a higher incidence of autism in boys than in girls. Hyperpeptidemia and increased blood–brain barrier permeability may cause accumulation of BCM7 in the blood and the brain, leading to the development of ASD [24,26]. We did not observe differences in BCM7 concentration in urine (Figure 1B), whereas Sokolov et al. [17] demonstrated that autistic children have significantly higher levels of urine BCM7 than healthy children, and the severity of autistic symptoms were correlated with concentrations of the peptide in the urine. These authors suggested that peptiduria in autistic children is the potential defect in their proteolytic and/or peptide excretion systems, consistent with previous studies that infants with delayed psychomotor development had elevated levels of the postprandial bovine BCM7 compared with a healthy control group. Chronic exposure to elevated levels of bovine casomorphins may contribute to disorders during early child development, also including autistic disorders, because these peptides interact with opioid and serotonin receptors, the known modulators of synaptogenesis [17,24]. Opioid peptides alter not only the mechanism of neuromodulation in the central nervous system (CNS), but also trigger inflammation and food allergy [12,36]. It is known that subcutaneous injection of BCM7 causes local pseudo-allergic reactions, masts cell degranulation, and histamine secretion even in healthy children [37]. β-casomorphin-5 may cause mast cells degranulation in mice, confirming the nature of the allergenic potential of this peptide [38]. Consequently, consumption of bovine milk containing BCM7 may induce inflammatory response in intestine by activating Th2 pathway [36], which was described in our previous research [12]. According to this approach, BCM7 consumption by autistic children induces gastrointestinal problems, such as abnormalities of the bowel mucosa, dysfunctions associated with intestine permeability, and changes in the gut microbiota [39]. High-protein diet may result in increased BCM7 concentration in the serum and urine of autistic children (Figure 1A,B). Consequently, milk-derived opioid peptides may reduce the uptake of cysteine, resulting in a decrease in glutathione synthesis and availability of the methyl donor S-adenosylmethionine (SAM). The decrease in SAM translates into effects on global DNA methylation, with epigenetic consequences, which alter neurological disorders such as autism [40]. Therefore avoiding milk-derived opioid peptides may lead to reduced peptiduria in children with autism and improves health, including silenced autoimmunity, improved emotional state and the opportunity to establish social relationships [24,29,41,42].

DPPIV is involved in immune response and nonspecific inflammation processes and its decreased activity is generally associated with impaired immune status [25]. While we did not observe significant difference in serum DPPIV activity between autistic group and control group (Figure 2A), deficiency and/or low enzymatic activity of DPPIV were suggested as possible causes for the presence of elevated levels of opioids in patients with autism, subsequently worsening autistic symptoms [23,43]. It has been reported, that DPPIV activity in women is slightly lower than in men [44], which was observed in the ASD group (Figure 2A). We demonstrated that female autistic children have lower levels of DPPIV activity than males (*p* < 0.01), whereas there were no significant differences between genders in control group (Figure 2B). This dependence is difficult to explain, but in our opinion its mechanisms should be sought in the biological role of DPPIV. It is known that DPPIV inactivates glucagon-like peptide (GLP)-1, and glucose-dependent insulinotropic peptide (GIP) exert pivotal functions on metabolic homeostasis such as glucose-dependent insulin secretion or suppression of excessive glucagon secretion. DPPIV causes degradation of milk-derived opioid peptides, stromal cell-derived factor 1 (SDF-1), substance P, and neuropeptide Y (NPY), and also participates in various processes, including immune stimulation, binding to extracellular matrix, and lipid accumulation [45].

Biological function of DPPIV is revealed not only by its enzymatic activity, but also by the concentration in the serum. Bashir and Laila [46] concluded that autistic patients have lower levels of plasma DPPIV than control group, but DPPIV content was not correlated to the severity of autism, according to CARS scoring results. Another authors did not find any defects in DPPIV in the blood of 11 autistic children [47]. We demonstrated that concentration of serum DPPIV is significantly higher (*p* < 0.01) in ASD children compared to the control group (Figure 2B). Constantly, there was also significant difference between ASD and control girls (*p* < 0.05) and ASD and control boys (*p* < 0.01). It should be noted that the highest DPPIV content was correlated with the highest serum BCM7 concentration among ASD girls. This dependence is clear to explain, because DPPIV is the only one enzyme that is able to hydrolyze the BCM7 and increased expression of DPPIV is the obvious response to the presence of BCM in blood (Figure 1A, Figure 2B and Figure 3). 

The presence of DPPIV was confirmed on the surface of T lymphocytes, simultaneously describing its abnormal expression in patients with various diseases [12,25], but information about changes in the expression of membrane forms DPPIV are still limited and require further research. It is known that BCM7 circulating in the bloodstream, can influence of the expression of DPPIV triggering a number of pathological mechanisms as inflammation and cytokine secretion. Impact of food ingredients on human physiological mechanisms is difficult to estimate in in vivo conditions, so we decided to use PBMCs as a model the human immune system. We confirmed the presence of BCM7 in extract obtained from hydrolysed bovine milk (60 ng/mL) and then we incubated PBMCs with this sample to estimate if milk-derived opioid peptides alter DPPIV gene expression on immune cells. While the carried out analysis has not revealed significant changes, a tendency to increase DPPIV gene expression in ASD girls was observed (Figure 3). Cieślińska et al. [33] indicated correlation in DPPIV gene expression under the influence of BCM7 and hydrolyzed milk between healthy and ASD-affected children, but only with genotype GG (polymorphism in DPPIV gene: rs7608798). Our previous studies revealed that PBMCs DPPIV gene expression was 1.5–2.5 times lower after BCM7 and hydrolyzed milk stimulation in patients with atopic dermatitis compared to the control group [12]. Therefore, changes in DPPIV gene expression should be considered as a potential factor with different biological effects depending on the studied disease.

The casein-free diet and avoidance of milk-derived opioid peptides are crucial in regulation of the DPPIV content and activity in patients with ASD. In autistic children we noted negative correlation between DPPIV activity and BCM7 concentration, and positive correlation between DPPIV concentration and DPPIV activity in serum. We observed positive correlation in both DPPIV activity and BCM7 concentration, and DPPIV concentration and DPPIV activity in serum among the control group (Figure 4A,B). According to gender positive correlation was found between DPPIV activity and DPPIV concentration in serum of ASD boys (Figure 5). In healthy girls, we noted a positive correlation between DPPIV activity and BCM7 concentration in serum, and DPPIV activity and DPPIV concentration in serum. (Figure 5). An interesting result of the present study was correlation between DPPIV content and BCM7 concentration in serum from girls with ASD. We suggest, that despite higher DPPIV content in serum, and its enzymatic activity per amount of protein was actually lower in autistic girls than in the other groups. The higher levels of protein could compensate for reduced overall activity of the enzyme. The overall difference between individual parameters between girls and boys seems to be interesting, it requires further research and should be considered at the level of sex hormones. Boys are subjected to higher testosterone activity than girls, which can affect brain function and anxiety in social situations. In turn, girls are subjected to a higher concentration of oxytocin, which facilitates social behavior and correlations. The effects of these hormones are revealed in fetal life and may affect the severity of autistic symptoms in later life [48,49,50]. Moreover, it has been observed that TSH level is about 31% lower in ASD boys in comparison to healthy ones [51]. Thyroid hormones are essential for brain maturation and function throughout life. Thyroid hormone deficiency, even for short periods, may lead to irreversible brain damage, the consequences of which depends on the specific timing of onset and duration of thyroid hormone deficiency [52]. 

To the best of our knowledge, this is the first study that examines the influence of BCM7 on the role of DPPIV in populations of healthy children and those diagnosed with autism spectrum disorder. The inspiration for this study emanated from clinical experience of the role daily diet in relieving the symptoms of autism. This paper considers the role of opioid peptides and DPPIV as potential factors in determining the pathogenesis of autism in aspect of BCM7 biological activity and recognizes numerous reports about the effectiveness of elimination diets (casein free) in the treatment of children with neurological disorders. However, this issue requires further investigation.

## Figures and Tables

**Table 1:** Primers used in Real-Time PCR for the analysis of DPP IV gene expression.

Gene	Forward Primer	Revers Primer	Annealing Temperature	Base Pairs
ACTB NM-001101.3	5′-TCC CTG GAG GAA GAG CTA CGA-3′	5′-AGC ACT GTG TTG GCG TAC G-3	60 °C	194 bp
DPPIV NM-001935	5′-GAA TTA TCC GGT CGG GTT TT-3′	5′-GTG ACA TCA CTG CCC ACA TC-3′	60 °C	189 bp
